# Methicillin-Resistant* Staphylococcus aureus* Recovered from Healthcare- and Community-Associated Infections in Egypt

**DOI:** 10.1155/2016/5751785

**Published:** 2016-06-28

**Authors:** Mohamed Abdel-Maksoud, Mona El-Shokry, Ghada Ismail, Soad Hafez, Amani El-Kholy, Ehab Attia, Maha Talaat

**Affiliations:** ^1^US Naval Medical Research Unit No. 3, Cairo 11517, Egypt; ^2^Global Disease Detection Center, US Centers for Disease Control and Prevention, Cairo, Egypt; ^3^Ain Shams University, Cairo 11566, Egypt; ^4^Alexandria University, Alexandria 21599, Egypt; ^5^Cairo University, Cairo 12316, Egypt; ^6^Ministry of Health and Population, Cairo 11516, Egypt

## Abstract

*Background.* Methicillin-resistant* Staphylococcus aureus* (MRSA) has created significant epidemiological, infection-control, and therapeutic management challenges during the past three decades.* Aim.* To analyze the pattern of resistance of healthcare- and community-associated MRSA in Egypt and the trend of resistance of HA-MRSA over time (2005–2013).* Methods.* MRSA isolates were recovered from healthcare-associated (HA) and community-associated (CA)* Staphylococcus aureus* (*S. aureus*) infections. They were tested against 11 antimicrobial discs and the minimal inhibitory concentration (MIC) of vancomycin was determined. Inducible clindamycin resistance (iMLSB) was also screened using D-test.* Findings.* Of 631* S. aureus*, MRSA was identified in 343 (76.6%) and 21 (11.5%) of HA and CA* S. aureus* isolates, respectively. The proportion of HA-MRSA increased significantly from 48.6% in 2005 to 86.8% in 2013 (*p* value < 0.001). Multidrug resistance (MDR) was observed in 85.8% of HA-MRSA and 48.6% of CA-MRSA. Vancomycin intermediate resistant* S. aureus* (VISA) was detected in 1.2% of HA-MRSA and none was detected in CA-MRSA. Among HA-MRSA strains, 5.3% showed iMLSB compared to 9.5% among CA-MRSA.* Conclusion.* The upsurge of the prevalence rates of HA-MRSA over time is alarming and urges for an effective infection control strategy and continuous monitoring of antimicrobial use.

## 1. Introduction

Methicillin-resistant* Staphylococcus aureus* (MRSA) infection is a significant contributor to morbidity and mortality in hospitalized patients worldwide and has been associated with several hospital outbreaks since the late 1970s [1]. Compounding the problem of healthcare-associated MRSA (HA-MRSA) is the growing prevalence of community-associated MRSA (CA-MRSA) raising the challenge of selecting the optimum therapy for both HA and CA MRSA infections [2].

MRSA rates among* S. aureus* clinical isolates scored the highest in Egypt compared to other African countries and to southern and eastern Mediterranean countries [3, 4].

Vancomycin has been the cornerstone for treating patients with HA-MRSA infection over the last 20 years [5]. Conceivably, treatment failure in cases of MRSA infections was mainly attributed to phenotypes with reduced susceptibility to vancomycin (RSV) which have brought vancomycin's utility into question [6]. Clindamycin, a member of macrolide-lincosamide-streptogramin B (MLSB) antibiotics, has surfaced as an efficient alternative for MRSA infections due to its special pharmacokinetic properties especially for penicillin allergic patients [7]. Consequently, inducible resistance to MLSB antibiotics has emerged worldwide necessitating the need to detect such resistance by a simple D-test on a routine basis [8].

The objective of this study was to analyze the pattern of resistance of healthcare- and community-associated MRSA in Egypt and to determine the trend of resistance of HA-MRSA over time (2005 through 2013).

*Data Analysis*. Statistical analysis was performed using *Z*-score test for two population proportions.

A *p* value < 0.05 was considered statistically significant.

## 2. Materials and Methods

This is an active prospective cohort study which took place in 12 hospitals in Egypt from 2005 to 2013. Healthcare-associated* S. aureus* (HA-*S. aureus*) isolates were recovered from patients acquiring infections after ≥3 calendar days of an admission to a health facility [9].

*S. aureus* strains were isolated from pus (12), wound swabs (23), urine (19), blood (135), bronchoalveolar lavage (123), endotracheal tube (6), sputum (56), and unknown sources (74).

Community-associated* S. aureus* (CA-*S. aureus*) isolates were collected through a sentinel surveillance program for acute febrile illnesses implemented in 12 infectious disease hospitals in Egypt from 2005 to 2013. These isolates were recovered from patients presenting with clinical symptoms <3 calendar days of an admission to a health facility indicating community-associated transmission [9]. They were isolated from blood (144) and CSF (39).

All isolates were shipped to the US Naval Medical Research Unit No. 3 (NAMRU-3) laboratories for identification and susceptibility testing to ensure the standardization of the techniques used and unifying the antimicrobial discs for susceptibility testing in concordance with Clinical Laboratory Standards Institute (CLSI) guidelines.* S. aureus* ATCC 25923 was used to verify the quality and accuracy of testing procedures [10, 11].

MRSA strains were tested against 11 antimicrobial discs (Becton Dickinson and Company, Sparks, MD, USA). The antibiotics used and their disk potencies are shown in Table 1.

Strains showing reduced susceptibility to vancomycin (RSV) are strains with MIC levels ≥2 mg/mL, while strains were confirmed to be VISA (vancomycin intermediate* S. aureus*) or VRSA (vancomycin resistant* S. aureus*) when vancomycin MIC levels were 4–8 *μ*g/mL and >8 *μ*g/mL, respectively [11].

### 2.1. The Double Disc Susceptibility Test (D-Test)

The presence of inducible clindamycin resistance (iMLSB) was sought in MRSA isolates that were erythromycin resistant and clindamycin sensitive (ER-R and CL-S) using the D-test [10].

## 3. Results

A total of 631* S. aureus* isolates were collected between 2005 and 2013. Of these, 448 (71%) were HA-*S. aureus* and 183 (29%) were CA-*S. aureus*. MRSA constituted 76.6% (343/448) of HA-MRSA and 11.5% (21/183) of CA-MRSA.

Resistance of HA-MRSA isolates to 11 antimicrobial discs was considerably high for most antibiotics used in the panel ranging from 55.6% to 100%, while resistance to rifampicin and trimethoprim-sulfamethoxazole was relatively lower (20.4% and 17.2%, resp.) (Table 1). On the other hand, CA-MRSA isolates showed a wider range of susceptibility to most antibiotics with resistance rates ranging from 9% to 20%, except for tetracycline (85%) and penicillin and ampicillin (100% each). MDR of HA-MRSA was significantly higher compared to CA-MRSA (85.7% and 47.6%, resp.).

The rate of HA-MRSA has significantly increased from 48.6% in 2005 to 86.8% in 2013. During the same time period, the proportion of HA-*S. aureus* with RSV (MIC levels ≥ 2 mg/mL) has significantly increased from 4.2% (3/72) in 2005 to 25.8% (51/197) in 2013 (*p* < 0.001).

Susceptibility to vancomycin was detected in 80.2% (275/343) of HA-MRSA and 95.2% (20/21) of CA-MRSA. VISA was detected in 1.2% (4/343) of HA-MRSA while none of CA-MRSA strains showed VISA pattern. VRSA was not detected in either HA-MRSA or CA-MRSA (Table 2).

A total of 29 HA-MRSA and three CA-MRSA isolates displaying CL-S and ER-R phenotype were tested using D-test. iMLSB (D-test positive) was identified in 5.3% (18/343) of HA-MRSA strains and in 9.5% (2/21) of CA-MRSA strains (Table 3).

## 4. Discussion

In the present study, prevalence of MRSA was higher among HA-*S. aureus* strains compared to CA-*S. aureus* strains. In support of this, other prospective surveillance studies conducted in Egypt reported similar findings [12, 13]. Meanwhile, reports emanating from Middle East countries also revealed increasing rates in the incidence of MRSA: in Saudi Arabia (77.5%) [14] and in Libya (54–68%) [15]. The compelling finding in this study was the substantial increase in MRSA prevalence over time. The prevalence rate of HA-MRSA has almost doubled from 48.6% to 86.8% between 2005 and 2013. Similar trends were also reported from a similar study conducted in Lebanon [16]. In contrast, successful attempts to reduce MRSA rates were also reported from USA and Israel through implementing a multimodel intervention including active surveillance, contact isolation, monitoring, and universal decolonization of patients in intensive care units [17, 18].

HA-MRSA demonstrated resistance to most antimicrobials used in this study with rates ranging from 55.6% to 100%. On the contrary, low resistance rates to rifampicin and trimethoprim-sulfamethoxazole (SXT) (20.4% and 17.2%, resp.) were observed. The high MDR of HA-MRSA isolates (85%) shown was similar to data reported from other developing countries, for example, Iran [19] and Sudan [20]. High rate of resistance could be explained by the response of the MRSA strains to the selection pressure created by their constant exposure to antibiotics used in hospital settings [20]. However, low resistance rates to rifampicin and trimethoprim-sulfamethoxazole (SXT) (20.4% and 17.2%, resp.) might be attributed to the limited use of rifampicin and SXT by Egyptian clinicians. This explanation is supported by two studies analyzing the rationale of antibiotic use in Egypt. The studies reported that beta lactam, macrolides, and cephalosporins antibiotics are the most common antibiotics prescribed for treating healthcare-associated infections in Egyptian hospitals [21, 22].

In the current years, the problem of MRSA has been aggravated by the spread of these resistant pathogens into the community. The prevalence of CA-MRSA (11.5%) in the present study is in concordance with the findings reported in other studies conducted in Egypt [23], Saudi Arabia [14], and India [24]. Resistant microorganisms from the community have been a major concern worldwide due to their rapid emergence and their potential to cause serious infections [14]. The rate of CA-MRSA in Egypt is mostly attributed to self-medication with antibiotics for mild bacterial or viral infections, where a total of 23.3 to 60% of antibiotics were received without physician prescription [25].

CA-MRSA has a wider spectrum of susceptibility compared to HA-MRSA with MDR rates of 47.76% and 85.7%, respectively. In support of this, studies from USA and India have reported that occurrence of MDR-MRSA strains was more prevalent in HA-MRSA than CA-MRSA indicating that HA-MRSA strains may be the important reservoirs of MDR strains, but now multidrug resistance is being slowly acquired by CA-MRSA strains [2, 24]. The present study revealed that more than 90% of the CA-MRSA strains were susceptible to gentamycin, ciprofloxacin, and clindamycin, suggesting that these drugs could provide a better option to treat CA-MRSA infections.

Reduced susceptibility to vancomycin has shown a continuous rise from 4.2% in 2005 to 25.8% in 2013 for HA-MRSA and the prevalence of intermediate resistance (VISA) was 1.2% (4/343) of HA-MRSA which is similar to the rates reported from Egypt [26] and other developing countries, for example, Iran [27] and Sudan [20], and higher than the rate reported from Japan [28].

The emergence and spread of resistance to vancomycin are a threat to the already challenging therapy of MRSA and raise an alarming situation to the clinicians in hospital as well as in community. Alternative therapies should be considered where vancomycin MIC is >1 *μ*g/mL to avoid treatment failure [27]. Clindamycin is one of the options that could be used for treating patients with either HA-MRSA or CA-MRSA. Different rates of inducible clindamycin resistance were reported from other countries, in USA and in India [29, 30].

In light of the previous data, reporting MRSA as susceptible to clindamycin without checking for inducible resistance using D-test may result in institution of inappropriate clindamycin therapy. On the other hand, negative result for inducible clindamycin resistance confirms clindamycin susceptibility and provides a very good therapeutic option [29].

The results of the study indicate the importance of developing policies and regulations for antibiotic use at the country level, implementing antibiotic stewardship programs to promote appropriate use of antibiotics, and increasing the awareness of clinicians and the public on rational use of antibiotics. In light of the restricted range of antibiotics available for the treatment of MRSA and the known limitations of vancomycin, clindamycin should be considered as an alternative for the management of serious MRSA infections sensitive to clindamycin. Laboratories should start considering using a D-test for diagnosis of inducible clindamycin resistance to avoid treatment failure. Enhancing infection prevention and control programs to contain HA-MRSA is crucial for Egypt.

## Figures and Tables

**Table 1:** Resistance of MRSA strains to antimicrobial discs.

	HA-MRSA (*n* = 343)	CA-MRSA (*n* = 183)
	Number (%)	Number (%)
Ampicillin (10 *μ*g)	343 (100)	21 (100)
Penicillin (10 *μ*g)	343 (100)	21 (100)
Tetracycline (30 *μ*g)	290 (84.5)	18 (85.7)
Gentamicin (10 *μ*g)	277 (80.7)	2 (9.5)
Ciprofloxacin (5 *μ*g)	242 (70.5)	2 (9.5)
Erythromycin (15 *μ*g)	221 (64.4)	4 (19)
Clindamycin (2 *μ*g)	191 (55.6)	0 (0)
Rifampicin (5 *μ*g)	70 (20.4)	3 (14.2)
SXT^*∗*^ (1.25 *μ*g/23.75 *μ*g)	59 (17.2)	4 (19)
Vancomycin E-test	0 (0)	0 (0)

^*∗*^SXT: trimethoprim-sulfamethoxazole.

**Table 2:** Minimum inhibitory concentration (MIC) levels of vancomycin amongst MRSA strains.

MIC (*µ*g/mL)	HA-MRSA (*n* = 343)		CA-MRSA (*n* = 21)	
	Number	%	Number	%
<2^*∗*^	275	80.2	20	95
3	64	18.6	1	4.7
4^*∗∗*^	4	1.2	0	0

HA: healthcare-associated; CA: community-associated; MRSA: methicillin-resistant *Staphylococcus aureus*; ^*∗*^<2 *µ*g/mL: susceptible to vancomycin; ^*∗∗*^4 *µ*g/mL: VISA.

**Table 3:** D-test for inducible clindamycin resistance.

Phenotype	HA-MRSA (*n* = 343)		CA-MRSA (*n* = 21)	
	Number	%	Number	%
ER-R, CL-S (D−ve)	11	3.2	1	4.8
ER-R, CL-S (D+ve)	18	5.3	2	9.5

HA: healthcare-associated; CA: community-associated; MRSA: methicillin-resistant *Staphylococcus aureus*; ER: erythromycin; CL: clindamycin; R: resistant; S: sensitive.
